# Cross-cultural comparison of mental illness stigma : A Multinational Population-Based Study from 16 Arab Countries and 10,036 Individuals

**DOI:** 10.1192/j.eurpsy.2023.745

**Published:** 2023-07-19

**Authors:** M. Stambouli, F. Fekih Romdhane, F. Ghrissi, A. Jaoua, S. Ellini, M. Cheour

**Affiliations:** Psychiatry department E, Razi hospital, Manouba, Tunisia

## Abstract

**Introduction:**

Attitudes toward people with mental disorders in Arab countries have undergone huge transformations throughout history. Stigmatization of the mentally ill has been a long tradition in our communities. The public’s views have evolved since then, however, little is known about the current situation regarding mental illness stigma in our context.

**Objectives:**

Explore attitudes towards mental illness and mental health knowledge in Arab countries.

**Methods:**

We carried out a multinational cross-sectional study using online self-administered surveys in the Arabic language from June to November 2021 across 16 Arab countries.The Community Attitudes toward the Mentally Ill scale,the Mental Health Knowledge Schedule scale and the Attitudes Toward Seeking Professional Psychological Help Scale-Short Form were administered to participants from the general public.

**Results:**

The study sample was predominantly female (77%), married (41%), educated (89% with tertiary education), living in urban areas (85%), with a mean age of 29.6 ± 10.8 years.

Based on the CAMI, MAKS, and ATSPPH-SF total scores, 75^th^, 50^th,^ and 25^th^ percentile were considered as cut-off points for the high, medium, and low scores. We found that 26.5% exhibited stigmatizing attitudes towards people with mental illnesses, 31.7% had poor knowledge, and 28.0% hold negative attitudes toward help-seeking. Regarding attitudes toward mental illness, the highest mean score was on the social restrictiveness subscale (35.1 ± 5.6), reflecting the lowest amounts of stigma in this dimension; while the lowest mean score was on the Authoritarianism subscale (32.0 ± 4.6).

We found a significant difference between countries regarding attitudes (*F*=194.8, p<.001) and knowledge (*F*=88.7, p<.001).

**Conclusions:**

Although much scientific progress has been made in the fields of diagnosing and treating mental illness, at a societal level the stigmatization of mental illness is still an important societal problem. The general population is largely ignorant about mental disorders, and fear of the mentally ill remains prevalent.

**Disclosure of Interest:**

None Declared

